# Roadmap to the Digital Transformation of Animal Health Data

**DOI:** 10.3389/fvets.2017.00123

**Published:** 2017-08-23

**Authors:** Jim Bracken

**Affiliations:** ^1^GS1 Global Office, Brussels, Belgium

**Keywords:** standards, GS1, livestock production, digitized animal welfare, antimicrobials

## The Context

The use of antimicrobials in livestock production provides a basis to improve animal health and productivity which, in turn, contributes to food security, food safety, animal welfare, protection of livelihoods, and animal resources. However, there is increasing concern about levels of antimicrobial resistance in bacteria isolated from human, animal, food, and environmental samples and how this relates to the use of antimicrobials in livestock production.

The reality is that both the quantity and quality of data available on the usage of antimicrobials in livestock production is grossly inadequate. As a consequence, it is virtually impossible to assess the extent of overuse of antimicrobials in the treatment of livestock production. Equally, the pharmaceutical industry has little or no data on what percentage of the animal medicines, which are sold are actually administered, nor does it have any information in terms of treatment versus outcome.

Although government regulations in many countries require the recording of medicines administered to food producing animals, these are largely in the form of manually maintained drug records. Apart from the additional workload, which this imposes on farmers these data are not readily available for further analysis. In order to tackle the many challenges from antimicrobial resistance, it is essential to have real-time accurate data about antibiotic use in the treatment of animals.

The best solution would be to have such data captured at the point of treatment of the animal(s) and stored in a digital drug record. This would facilitate not only the sharing of such important data but would also enable further analysis for animal welfare and other purposes. This is possible using open global standards for the recording, storing, and sharing of such critical animal welfare data and should be an integral part of the animal traceability system.

## What are the Drivers for the Move to Digital, Real-Time Data on the Treatment of Animal Disease?

- Public health—the need to tackle the increasing problems of health acquired infections caused by the prevalence of antibiotic resistant bacteria.
- Animal welfare—the need to treat animals with more appropriate levels of medication.
- Consumer trust—consumers need to have trust in the food supply chain, this is evident from the recent Horsegate scandal and the bovine spongiform encephalopathy outbreak.
- Sustainable agriculture—the need to reduce the impact of overuse of antibiotics on the soil and watercourses of farms.
- Provenance required by brands and retailers—as buyers, they are seeking to have greater assurance about the source of the food products, which they are selling to their customers.
- Technology—the Internet of Things (IOT) makes it possible to connect data on individual objects such as animals to systems for recording and reporting on their treatment and welfare.

## How Can Digitized Animal Welfare Records be Delivered?

**Figure 1:**
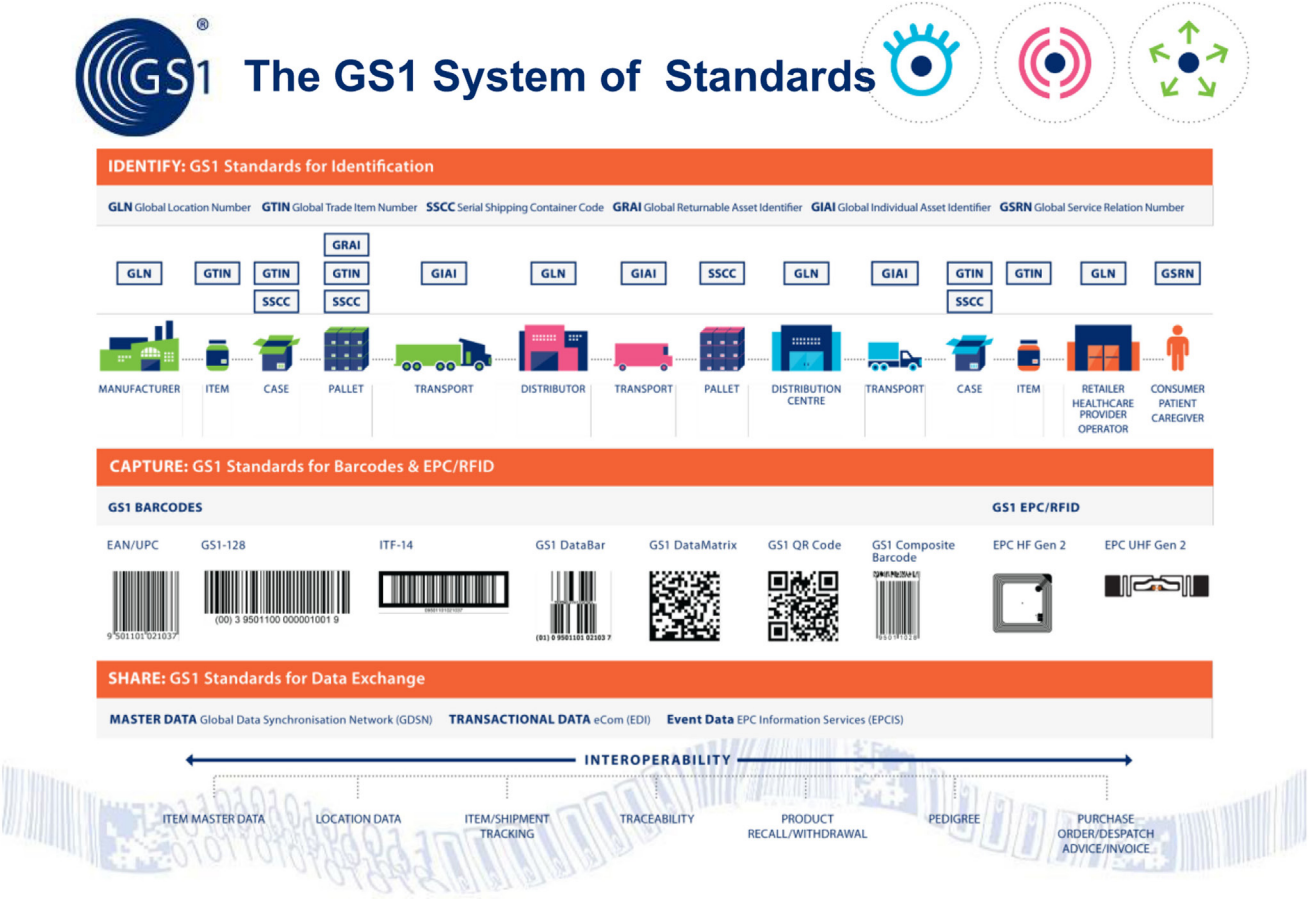
How GS1’s global standards work to move data between the various supply chain partners, thereby linking the physical flow of goods to the electronic data associated with them.

First, we need to start by using existing automatic data capture standards provided by GS1 (Figure 1). These are already used by more than 1.3 million companies operating in the food and some 20 other industry sectors worldwide. Indeed, the veterinary pharmaceutical manufacturers already mark their products with GS1 2D barcodes, which contain the product ID, the batch number, and expiry date. This means that veterinary surgeons/farmers could scan and record details of the medication administered to each animal.

Second, these data need to be stored in an animal’s health record, again, there are global standards already in place for human health records (HL7), which could form the basis of an animal health record standard.

Third, data on animals/batches of animals could be shared between trusted parties across the food supply chain using the IOT. GS1’s open standard—the Electronic Product Code Information System (EPCIS) enables this.

As for animal health records, capturing details of medication at point of administration to an animal would ensure greater accuracy of its health record. This also means that analysis can be carried out to compare treatment versus outcome, and this will not only help to improve medication regimes but will also provide invaluable data for other stakeholders, especially the veterinary pharma sector. In order to ensure interoperability of information and communication technology (ICT) systems and solutions, it is essential that a global standard is used for recording medication and disease data, given that HL7 already exists for this purpose for human health records, it seems obvious that this would be the most suitable solution to adopt.

This exact approach was very successfully implemented in the treatment of Irish hemophilia patients resulting not only in a complete traceability solution but significant improvement in terms of patient safety. The solution is centered on an electronic patient record and uses a mobile phone app to scan all medication administered along with recording some clinical data. This real-time data collection enables clinicians to be proactive in the management of a patient’s condition.

Similarly, a beef traceability solution was put in place covering the process from farm to fork, although initially designed to meet the batch traceability requirements of EC 1760 and the EU Food Law EC178, the updated system provides a one-to-one link between all primals produced from each animal. This means that if a customer has a complaint about an individual steak, then its provenance can immediately be checked back and any remedial action necessary can be taken. The system is based on the scanning of standardized labels placed on the primals, so when the retail butcher is preparing the meat for prepack or serve-over, the associated data are linked to the prepack label and ultimately to the customer’s till receipt. A major German food retailer is using an EPCIS solution to provide customers with assurance on the traceability and sustainability of their meat and fish.

In conclusion, it is true to say that by leveraging the use of ICT and the IOT, it is eminently possible to make a transformational change to the way in which an animal’s/batch of animals’ medication history can be recorded and shared between trusted parties. Such a move can only help in the move toward more sustainable food production in compliance with the UN Sustainable Development Goals. Last, from the writer’s experience, traceability solutions based on open global standards invariably produce cost savings and real return on investment.

## Author Contributions

This manuscript was written by JB based on his knowledge of supply chain management and his considerable experience in the implementation of traceability solutions in the food, health care, and cosmetic sectors.

## Conflict of Interest Statement

The author declares that the research was conducted in the absence of any commercial or financial relationships that could be construed as a potential conflict of interest.

